# SHARE – workpackage 5: evidence-based recommendations for diagnosis and treatment of kawasaki disease and henoch schönlein purpura

**DOI:** 10.1186/1546-0096-12-S1-P122

**Published:** 2014-09-17

**Authors:** Nienke de Graeff, Noortje Groot, Sylvia Kamphuis, Tadej Avcin, Brigitte Bader-Meunier, Pavla Dolezalova, Brian Feldman, Isabelle Kone-Paut, Pekka Lahdenne, Alberto Martini, Liza McCann, Clarissa Pilkington, Angelo Ravelli, Annet van Royen, Nico Wulffraat, Seza Ozen, Paul Brogan, Michael Beresford

**Affiliations:** 1 Wilhelmina Children's Hospital, Utrecht, Netherlands; 2 Alder Hey Children's Hospital, Liverpool, UK; 3 Sophia Children's Hospital, Erasmus Medical Centre, Rotterdam, Netherlands; 4 University Children's Hospital Ljubljana, Ljubljana, Slovenia; 5 Necker Hospital, Assistance Publique-Hôpitaux de Paris, Paris, France; 6 General University Hospital, Prague, Czech Republic; 7 The Hospital for Sick Children, Toronto, Canada; 8 Bicêtre University Hospital, Paris, France; 9 Hospital for Children and Adolescents, University of Helsinki, Helsinki, Finland; 10 Gaslini Children's Hospital, Genoa, Italy; 11 Alder Hey Children’s Hospital, Liverpool, UK; 12 Great Ormond Street Hospital for Children, London, UK; 13 Dept. of Pediatric Rheumatology, Hacettepe University, Ankara, Turkey; 14 University of Liverpool, Liverpool, UK

## Introduction

Kawasaki Disease (KD) and Henoch Schönlein Purpura (HSP) are paediatric vasculitides that can lead to significant morbidity. Evidence-based guidelines are sparse and management is mostly based on physician experience. Consequently, treatment regimens differ throughout Europe. In 2012, a European initiative called SHARE (Single Hub and Access point for paediatric Rheumatology in Europe) was launched to optimize and disseminate guidelines for diagnosis and management for children and young adults with paediatric rheumatic diseases (PRD) such as KD and HSP within Europe.

## Objectives

To provide evidence-based recommendations for diagnosis and treatment of paediatric vasculitides, specifically KD and HSP.

## Methods

Evidence based recommendations were developed using evidence drawn from systematic reviews of the literature. An expert committee was formed consisting of paediatric rheumatologists from across Europe with expertise in vasculitis. Preliminary statements regarding recommendations on diagnosis and treatment of KD and HSP were developed. These recommendations were evaluated by the expert committee using an online survey. Those with less than 80% agreement in the online survey were reformulated. Subsequently, all recommendations were discussed at a consensus meeting in Genoa (Italy) in March 2014 using the nominal group technique [1]. Recommendations were accepted if more than 80% agreement was reached.

## Results

Evidence supporting recommendations for diagnosis and treatment was extracted from the literature. Subsequently, 53 statements on diagnosis and treatment were formulated based on this evidence and expert opinion and were evaluated in an online survey. After discussion of the statements at the consensus meeting, 29 recommendations for KD and 15 recommendations for HSP were accepted with more than 80% agreement during the meeting. Topics covered were criteria for diagnosis, referral of patients, clinical symptoms, useful laboratory investigations, imaging techniques and treatment.

## Conclusion

The SHARE initiative provides recommendations for diagnosis and treatment of paediatric vasculitides and thereby facilitates improvement and uniformity of care for patients throughout Europe. Currently, similar processes are ongoing to add additional recommendations on diagnosis and treatment where consensus to date has not been reached, as well as recommendations regarding the holistic care of patients. As a final result, SHARE will provide standards of minimal care for different PRDs, including KD and HSP, as well as more rare vasculitides (PAN, GPA, MPA, EGPA and TA).

## Disclosure of interest

None declared.
